# Trends and Prevalence of Psychotropic Medication Use in Children and Adolescents in the Period Between 2013 and 2023: A Systematic Review

**DOI:** 10.7759/cureus.55452

**Published:** 2024-03-03

**Authors:** Yasir Altuwairqi

**Affiliations:** 1 Psychiatry, College of Medicine, Taif University, Taif, SAU

**Keywords:** adolescents, children, psychiatric diseases, psychotropic medication, prevalence, trends

## Abstract

Mental health problems among children and adolescents are a significant global public health concern, with a prevalence of approximately 10-20%. Psychotropic medications, including stimulants, antipsychotics, antidepressants, and mood stabilizers, have been proven effective in treating various psychiatric disorders among children and adolescents. Despite the common use of these medications, they have various side effects and complications. This systematic review aimed to assess the trends and prevalence of psychotropic medication use among children and adolescents from 2013 to 2023. A comprehensive literature search was conducted in PubMed, Web of Science, Ovid, Scopus, and Cochrane databases using relevant keywords. Two independent researchers screened the studies for inclusion and exclusion criteria. Data were extracted using a Microsoft Excel spreadsheet (Microsoft Corporation, Redmond, WA), including information on study characteristics, participant demographics, psychiatric disorders, and psychotropic medications. The risk of bias assessment was performed using the ROBINS-I (Risk of Bias in Non-randomized Studies of Interventions) tool for non-randomized studies of interventions (NRSI) and Risk of Bias 2 (ROB2) for the randomized clinical trial. Data synthesis was conducted through a qualitative interpretation of the findings. A total of 52 papers were identified through the search, with 37 remaining after duplicate removal. After applying the inclusion and exclusion criteria, nine articles were considered suitable for the systematic review. A total of 9,034,109 patients suffered from several psychiatric diseases, such as autism, major depressive disorder, Down syndrome, attention-deficit/hyperactivity disorder, adjustment disorder, anxiety, bipolar disorder, conduct disorder, depression, personality disorder, psychotic disorder, tic disorder, pervasive developmental disorder, and disruptive behavior disorder. Stimulants showed a consistent prevalence rate over the years. Antidepressants, including selective serotonin reuptake inhibitors, have demonstrated variations over the years, with a substantial increase in 2015, followed by a decrease in subsequent years. In addition, antipsychotics, including atypical antipsychotics, have varied over the years; however, their use increased in 2023. Anticonvulsants and anxiolytics were also utilized, albeit at lower prevalence rates. This systematic review provides an overview of the trends and prevalence of psychotropic medication use among children and adolescents from 2013 to 2023. The prevalence of antipsychotic prescribing has shown fluctuations among different countries over the years, with a decline in recent years but a slight increase in 2023. Further research is warranted to explore the factors influencing these trends and to assess the long-term effectiveness and safety of psychotropic medications in children and adolescents.

## Introduction and background

Mental health disorders, also known as mental illnesses, are conditions characterized by a clinically significant disturbance in an individual's cognition, emotional regulation, or behavior and are usually associated with distress or impairment in essential functioning areas [1].

Mental health disorders are common among young adults in their 20s and somewhat less common in their 30s and 40s [2]. Some of the most common mental health disorders in children and adolescents include adjustment disorders, disruptive disorders, attention-deficit/hyperactivity disorder (ADHD), anxiety and mood disorders, attachment disorders, and autism [3]. Child and adolescent psychiatry used to be considered a part of adult psychiatry. However, in adult psychiatry, patients with schizophrenia are usually the focus, and their problems usually arise after puberty. Unfortunately, the problems that arise during their developmental stage are often overlooked. In child and adolescent psychiatry, the nature of diseases changes gradually. Diseases that usually develop in adulthood, such as schizophrenia, are rare in this stage, while diseases that usually develop in infants, such as persistent depressive disorder or ADHD, are very common [4].

In 2019, 301 million people were diagnosed with anxiety, and 280 million people suffered from depression. Among them, 58 million were children and adolescents affected by anxiety, and 23 million were children and adolescents affected by depression [5].

Psychotropic medications are a class of drugs that are used to treat mental health disorders [6]. These medications can be classified into several categories according to the primary therapeutic effects and the mental health disorders they are intended to treat [7]. The classifications of medications were antidepressants and anxiolytics, antipsychotics, and mood stabilizers [8]. There are various types of medications that are used to treat different mental health conditions. For instance, sertraline, escitalopram, and mirtazapine are commonly prescribed as antidepressants. Lithium, valproic acid, and lamotrigine are commonly used to stabilize mood. Selective serotonin reuptake inhibitors, benzodiazepines, and beta-blockers are some of the commonly used anti-anxiety and anti-depressant medications. Aripiprazole and asenapine are some of the commonly used antipsychotics. Amphetamine and atomoxetine are used in the treatment of ADHD [9].

According to a recent cohort study conducted on children and adolescents with type 1 diabetes, the dispensation of psychotropic medications, particularly hypnotics, ADHD medication, anxiolytics, and antidepressants, increased from 0.85% in 2009 to 3.84% in 2019 among children and from 2.72% in 2009 to 13.54% 2019 among adolescents [10].

Psychotropic medications, such as clozapine, haloperidol, olanzapine, phenothiazines, quetiapine, risperidone, ziprasidone, amitriptyline, clomipramine, and imipramine, are commonly used to treat mental health conditions. However, they may have various side effects and complications. These medications can lead to systemic side effects, such as metabolic diseases, cardiovascular diseases, and sexual dysfunction, along with other physical illnesses. The most commonly reported complications of these physical conditions include paralytic ileus, fecal impaction, bowel obstruction, and intestinal or bowel perforations [11]. Vision loss, blindness, and other eye-related issues associated with the use of antipsychotics and antidepressants are also considered significant concerns [12,13].

Psychotropic medications are often prescribed to children and adolescents with mental disorders. However, the increased use of psychotropic medications is a topic of debate among professionals and the public [14].

This systematic review aims to provide an in-depth analysis of recent trends and prevalence rates of psychotropic medication use among children and adolescents worldwide.

## Review

Methodology

This systematic review complied with established criteria (Preferred Reporting Items for Systematic Reviews and Meta-Analyses, PRISMA) [15].

Search Strategy

The systematic review was conducted in January and February 2024 through a thorough literature search of PubMed, Web of Science, Ovid, Scopus, and Cochrane databases using the keywords in the abstract and title: (trend AND psychotropic medication AND children). One researcher screened studies to select studies that matched the inclusion and exclusion criteria.

Inclusion and Exclusion Criteria

All papers assessing the trends and prevalence of psychotropic medication use for children and adolescents were included in the systematic review. The included studies were conducted in five countries: the United States, Canada, New Zealand, Denmark, and Australia. We excluded narrative reviews, systematic reviews, duplicated papers, studies published before 2013, studies with insufficient data or findings, studies with irrelevant findings, studies that include adults, studies that included the subjects’ ages ranged till 21 years, studies in a language other than English, and studies that are not free. If the studies involved data from before and after 2013, we considered data from 2013 and afterward.

Screening and Data Extraction

A reference manager was used to check the output of the search technique for duplication. First, the titles and abstracts of the relevant studies were screened. The author then examined relevant full-text papers and evaluated them for inclusion criteria. After that, the data were extracted using a Microsoft Excel spreadsheet (Microsoft Corporation, Redmond, WA). The data included authors, year of publication, country, study design, objective, sample size, participant characteristics, psychiatric disorder, and psychotropic medications.

Strategy for Data Synthesis

A summary table was created using data from relevant studies to provide a qualitative interpretation of the findings and study components.

Risk of Bias Assessment

In this systematic review, the risk of bias assessment was conducted by two authors among eight non-randomized studies of interventions (NRSI) and one randomized clinical trial. We used the ROBINS-I (Risk of Bias in Non-randomized Studies of Interventions) tool to assess NRSIs [16]. In addition, Risk of Bias 2 (ROB2) [17] was used to assess the randomized clinical trial. Two independent researchers conducted the assessments. The outcomes assessed were the prevalence and trends of psychotropic medications used among children and adolescents during 2013-2023. The judgment options were low, moderate, serious, and critical for ROBINS-I and low risk, high risk, some concerns, and no information for ROB2. The overall risk of bias was reached using signaling questions.

Results

**Figure 1:**
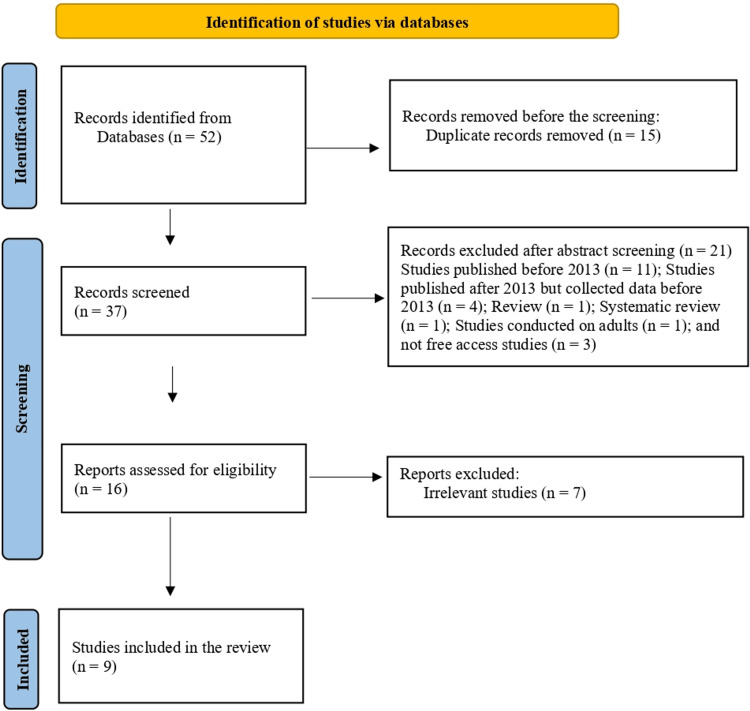
Flow diagram of study selection for the systematic review

A total of 52 papers were extracted through five databases (PubMed, Web of Science, Ovid, Scopus, and Cochrane), of which 15 were omitted as duplicates. Regarding the remaining 37 articles, 11 were excluded because they were published before 2013; four articles included data on psychotropic medications before 2013; one was a review article; one was a systematic review; one article included adult patients, and three were not free-access papers. Following screening and assessment, seven articles were irrelevant to the scope of the review. Nine articles were considered suitable for the systematic review (Figure 1).

Overview of the Included Studies

The included papers were published between 2014 and 2023 in different countries (United States, Canada, New Zealand, Denmark, and Australia). All studies focused on the prescribed psychotropic medications among children and adolescents. The study design varied among the included studies; six papers were retrospective descriptive studies, one was a randomized clinical trial, one was a descriptive study, and one was a longitudinal, multisite study. Across the nine studies, the sample sizes ranged from 196 to 7,426,926 subjects, with a total of 9,034,109 patients. In addition, the subjects’ ages ranged from 0 to 21 years. The patients suffered from several psychiatric diseases, such as autism, major depressive disorder, Down syndrome, ADHD, adjustment disorder, anxiety, bipolar disorder, conduct disorder, depression, personality disorder, psychotic disorder, tic disorder, pervasive developmental disorder, and disruptive behavior disorder. Particularly, three studies used samples of patients with autism, two represented a sample of subjects diagnosed with depressive disorders, two studies included patients with ADHD, and only one trial included patients with Down syndrome.

The psychotropic medications reported in included studies were antipsychotics, stimulants, non-stimulants, antidepressants, anticonvulsants, anxiolytics, mood stabilizers, amphetamine and dextroamphetamine, hypnotics/sedatives, and melatonin. All details are described in Table 1.

**Table 1:** Information excluded from the included studies

Authors, year	Objective	Study design	Sample size	Participant's characteristics	Psychiatric disorder	Psychotropic medications
Brenner et al. (2015) [18], United States	To examine mental health service use during the survey of outcomes following treatment for adolescent depression period	Randomized clinical trial	196	Age range: 12-17 years. Mean (SD): 17.8 (1.8) years. Gender: female: 56.1%; male: 43.9%	Major depressive disorder	Medication and/or psychotherapy: (58.7%); medication and psychotherapy: (30.1%); non-stimulant medication: (46.4%); antidepressants: (44.9%); antipsychotics: (8.2%); anxiolytics: (3.1%); mood stabilizers: (6.6%)
Downes et al. (2015) [19], United States	To examine the psychotropic medication use rate among children and adolescents with Down syndrome	Retrospective cohort from 2010 to 2013	832	Age range: 5-21 years. Gender: males: 55.1%; females: 44.9%	Down syndrome	Central nervous system stimulant: 5-11 years: 52 (9.6%); 12-21 years: 28 (7.6%). Selective serotonin reuptake inhibitor: 5-11 years: 22 (4.1); 12-21 years: 54 (14.7). Atypical antipsychotic: 5-11 years: 19 (3.5%); 12-21 years: 33 (9.0%). Alpha-adrenergic agonist: 5-11 years: 38 (7.0%); 12-21 years: 35 (9.5%). Others: 5-11 years: 92 (17%); 12-21 years: 92 (25%)
Pringsheim et al. (2019) [20], Canada	To identify the rate and trends of psychotropic drug prescribing among Canadian children in the period between 2010 and 2016	A retrospective comprehensive overview of psychotropic medication prescribing to Canadian children from 2012 to 2016	7,426,926	Less than 18 years	Attention deficit/hyperactivity disorder, adjustment disorder, anxiety, autism, bipolar disorder, conduct disorder, depression, personality disorder, psychotic disorder, and tic disorder	First-generation antipsychotics: 2013: 0.3%; 2014: 0.3%; 2015: 0.5%; 2016: 0.2%. Second-generation antipsychotics: 2013: 16.7%; 2014: 14.7%; 2015: 13.9%; 2016: 13.5%. Aripiprazole: 2013: 21.9%; 2014: 30.2%; 2015: 25.1%; 2016: 23.7%. Quetiapine: 2013: 27.5%; 2014: 18.5%; 2015: 16.2%; 2016: 17.0%. Risperidone: 2013: 41.5%; 2014: 43.8%; 2015: 52.1%; 2016: 52.9%. Selective serotonin reuptake inhibitor: 2013: 15.9%; 2014: 15.5%; 2015: 16.6%; 2016: 17.1%. Escitalopram: 2013: 18.1%; 2014: 16.5%; 2015: 12.8%; 2016: 15.8%. Fluoxetine: 2013: 37.4%; 2014: 55.0%; 2015: 43.3%; 2016: 43.2%. Sertraline: 2013: 14.6%; 2014: 9.1%; 2015: 20.6%; 2016: 19.9%. Serotonin-norepinephrine reuptake inhibitors: 2013: 2.2%; 2014: 2.5%; 2015: 2.1%; 2016: 2.0%. Psychostimulants: 2013: 54.7%; 2014: 52.3%; 2015: 52.6%; 2016: 53.4%. Lisdexamfetamine: 2013: 20.1%; 2014: 20.5%; 2015: 17.9%; 2016: 22.8%. Methylphenidate: 2013: 61.5%; 2014: 68.4%; 2015: 73.62%; 2016: 70.1%. Amphetamine and dextroamphetamine: 2013: 12.6%; 2014: 8.0%; 2015: 6.4%; 2016: 4.5%. Nonstimulants: 2013: 8.5%; 2014: 11.8%; 2015: 11.2%; 2016: 9.6%. Guanfacine: 2013: 4.8%; 2014: 30.1%; 2015: 35.1%; 2016: 30.2%. Clonidine: 2013: 36.6%; 2014: 27.5%; 2015: 26.6%; 2016: 27.1%. Atomoxetine: 2013: 58.6%; 2014: 42.4%; 2015: 38.2%; 2016: 42.7%
Barczyk et al. (2020) [21], New Zealand	To examine the psychotropic medication prescription frequency and trends for children and adolescents between 2008 and 2016	Retrospective study from 2008 to 2016	All prescriptions dispensed in New Zealand between 2008 and 2016	From 0 to 19 years	Not applicable	Any psychotropic medication: 2013: 1.99%; 2014: 2.1%; 2015: 2.22%; 2016: 2.36%. Antidepressants: 2013: 0.85%; 2014: 0.90%; 2015: 1%; 2016: 1.07%. Antipsychotics: 2013: 0.29%; 2014: 0.32%; 2015: 0.34%; 2016: 0.37%. Anxiolytics: 2013: 0.14%; 2014: 0.15%; 2015: 0.14%; 2016: 0.15%. Sedatives/hypnotics: 2013: 0.22%; 2014: 0.20%; 2015: 0.22%; 2016: 0.22%. Stimulants: 2013: 0.88%; 2014: 0.94%; 2015: 0.99%; 2016: 1.06%
Bushnell et al. (2021) [22], United States	To examine trends of annual use of antipsychotic medication among US young children (2-7 years) and to examine clinical and treatment characteristics of these children	A descriptive study from 2007 to 2017	2013: 4,981; 2014: 4,322; 2015: 2,964; 2016: 2,771; 2017: 2,501	Range: 2-7 years	Pervasive developmental disorder (38%). Disruptive behavior disorder (21%). Attention-deficit/hyperactivity disorder (18%)	A total of 301,311 antipsychotic prescriptions were prescribed from 2007 to 2017. Antipsychotic use in 2013: 0.23%; 2014: 0.20%; 2015: 0.18%; 2016: 0.17%; 2017: 0.17%. Types: Most medications prescribed were risperidone 0.75 mg/day (69%) or aripiprazole 5.0 mg/day (20%)
Klau et al. (2022) [23], Australia	To quantify prescribing psychotropic medications to children and adolescents trends in Australian primary care from 2011 to 2018	A retrospective cohort study from 2011 to 2018	537,371	Age: less than 19 years	-	Antipsychotics: 2013: 3.5%; 2014: 4.1%; 2015: 4.6%; 2016: 4.9%; 2017: 4.9%; 2018: 4.5%. Antidepressants: 2013: 20.9%; 2014: 22.3%; 2015: 22.7%; 2016: 24.4%; 2017: 25.5%; 2018: 24.2%. Attention-deficit hyperactivity disorder drugs: 2013: 6.8%; 2014: 7.7%; 2015: 8.1%; 2016: 8.6%; 2017: 9.8%; 2018: 10.4%. Anxiolytics: 2013: 3%; 2014: 2.9%; 2015: 3%; 2016: 3%; 2017: 3.3%; 2018: 2.5%. Hypnotics/sedatives: 2013: 1.9%; 2014: 1.9%; 2015: 1.7%; 2016: 1.7%; 2017: 1.6%; 2018: 1.2%. Melatonin: 2013: 4.2%; 2014: 5.9%; 2015: 7.4%; 2016: 9%; 2017: 11.1%; 2018: 11.8%.
Bliddal et al. (2023) [24], Denmark	To assess the rate of use of psychotropic medications and psychiatric disorders among Danish children, adolescents, and young adults during the COVID-19 outbreak	A population-based, descriptive cohort study from January 1, 2017, until June 30, 2022	108,840	Age range: 14-24 years. Gender: 54% females and 44% males	-	The overall rate of psychotropic medication use was 18% in treatment with hypnotics, antidepressants, and psychostimulants. The rate of medication use increased among subjects aged 12 to 17 years by 37%
Rast et al. (2023) [25], United States	To identify rates and patterns of psychotropic medication use among children and youth suffering from autism and enrolled in Medicaid	Retrospective descriptive study from 2008 to 2016	942,125	Age: 0-21 years	Autism	Any psychotropic medication: 2013: 48%; 2014: 47%; 2015: 45%; 2016: 44%. Antipsychotics: 2013: 27%; 2014: 26%; 2015: 24%; 2016: 22%. Stimulants: 2013: 20%; 2014: 21%; 2015: 20%; 2016: 20%. Antidepressants: 2013: 19%; 2014: 20%; 2015: 19%; 2016: 20%. Anticonvulsants: 2013: 14%; 2014: 14%; 2015: 14%; 2016: 14%. Anxiolytics: 2013: 2%; 2014: 2%; 2015: 2%; 2016: 2%
Shurtz et al. (2023) [26], United States	To assess the rate and type of psychotropic medications reported among a school-aged cohort of children with autism spectrum disorder	Longitudinal, multisite study	280	Age: mean (SD): 8.6 (1.7) years. Range: 6.0-11.6 years. Gender: male: 77.0%; female: 23.0%	Autism spectrum disorder	Psychotropic medications (N = 119): *Monotherapy (n = 60, 50.4%); polytherapy: (n = 59, 49.6%); melatonin (n = 53); stimulants (n = 45); selective serotonin reuptake inhibitors (n = 37); alpha agonists (n = 36); antipsychotics (n = 20)

Risk of Bias Assessment

**Figure 2:**
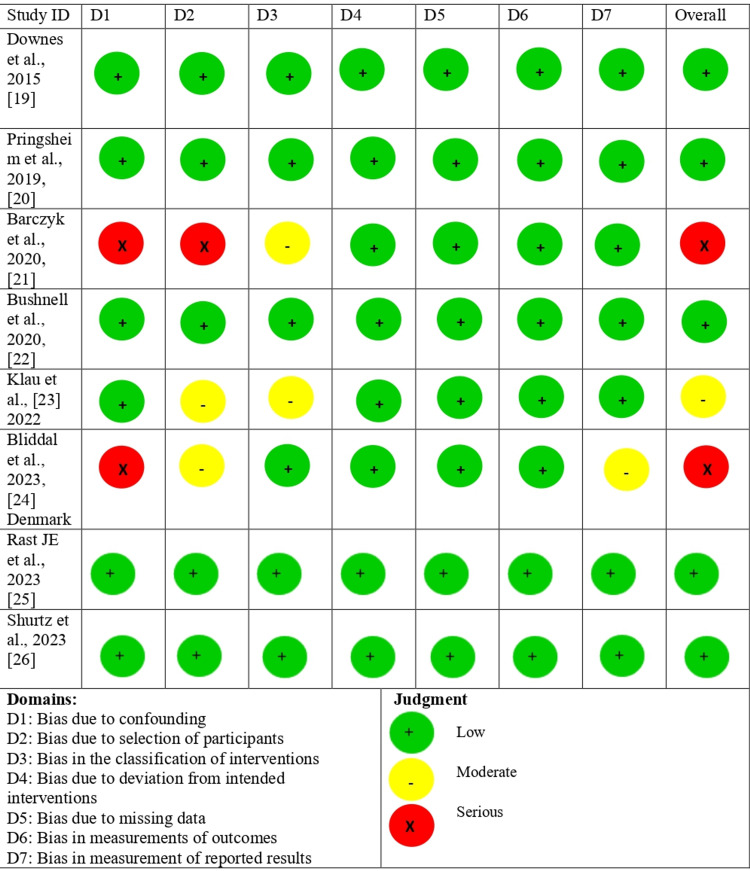
Robvis traffic light plot

**Figure 3:**
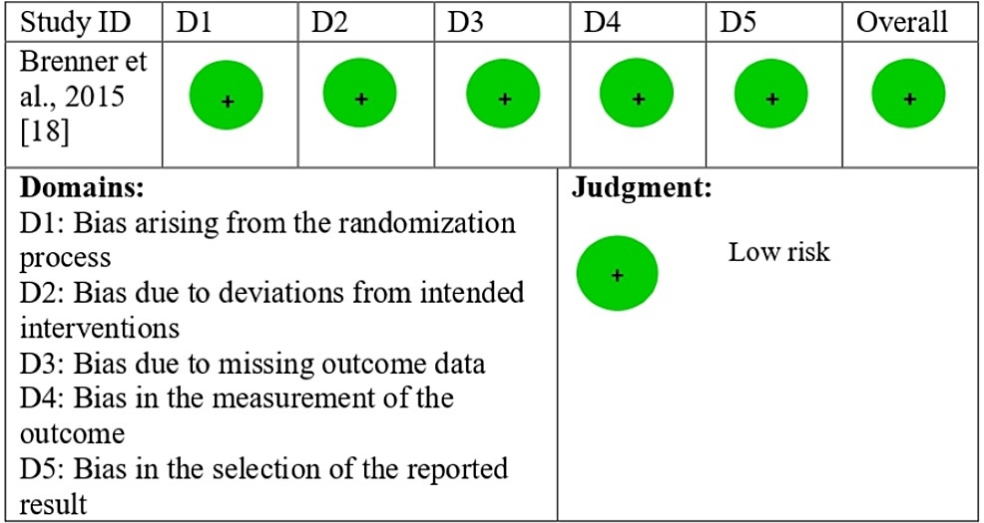
ROB2 traffic light plot ROB2: Risk of Bias 2.

The risk of bias revealed the overall quality of the included studies. Out of the nine studies, there was a moderate risk of bias in one study and a low risk of bias in two studies, according to the ROBINS-I tool (Figures 2, 3).

Discussion

Antipsychotic medications have been approved by the Food and Drug Administration (FDA) and the European Medicines Agency (EMA) for the treatment of specific psychiatric conditions such as schizophrenia, bipolar disorder, tic disorders, and severe irritability in autism spectrum disorders in children. However, these drugs are often prescribed off-label to children with other conditions such as conduct disorder, ADHD, anxiety, and depression [27-31]. This systematic review aimed to examine the trends and prevalence of psychotropic medication use in children and adolescents in the period between 2013 and 2023.

It is estimated that a significant number of children and adolescents worldwide experience mental health problems, with a prevalence of 10-20%. However, only a small proportion, approximately 5%, receive any form of mental health care. Psychotropic drugs such as stimulants, antipsychotics, antidepressants, and mood stabilizers have been established as effective treatments for various psychiatric disorders in this population, leading to their widespread use [32-34].

In the current review, the prevalence of antipsychotic prescribing has varied over the years among patients with diverse psychiatric disorders, with studies showing a range of percentages. In 2013, the prevalence ranged from 0.23% to 27% [20-23,25], while in 2014 and 2015, it ranged from 0.20% to 26% and 0.18% to 24%, respectively [18-23,25]. By 2016, the prevalence decreased to a range of 0.17% to 22% [20-23,25], and in 2017, it further declined to a range of 0.17% to 4.9% [22,23]. In 2018, the prevalence was reported as 4.5% [23]. On the other hand, a study conducted in 2022 found an increase in antipsychotic use, with approximately 7.14% of patients being prescribed these medications [26].

Regarding antipsychotic medications, stimulants, such as methylphenidate and amphetamines, are commonly prescribed for ADHD, making them the most frequently utilized psychotropic medications among children [35,36]. In our review, the prescribing rate of stimulants was similar over the years. In 2013, the range of prescriptions was 0.88% to 20%, followed by a similar rate in 2014 and 2015 (0.94%-21% and 0.99%-20%) [21,25]. In 2016, the range was from 0.99% to 20% among two studies [21,25]. In 2022, the prevalence of stimulant prescribing among children with autism spectrum disorder was reported as 16.1% [26].

In the United States, Australia, and Canada, the used antipsychotics were atypical [19,20,22,23]. In Canada, the atypical antipsychotics used were aripiprazole, quetiapine, and risperidone [20]. Similarly, in the United States, aripiprazole and risperidone were used, while in Australia, quetiapine, olanzapine, periciazine, lurasidone, and risperidone were used [22,23].

Antidepressant drugs are considered the primary treatment option for moderate to severe depression [37]. In recent years, there has been an increase in antidepressant consumption, primarily due to their long-term use rather than an escalation in the incidence and prevalence of the condition. Antidepressants are also recommended as first-line agents for generalized anxiety disorder (GAD), posttraumatic stress disorder (PTSD), and obsessive-compulsive disorder (OCD) in both children and adults [38,39].

According to the mentioned studies, in the United States, Australia, New Zealand, and Canada, the most commonly used antidepressants are selective serotonin reuptake inhibitors [18-23,26]. Fluoxetine is the preferred selective serotonin reuptake inhibitor in the United States, [18] while in Canada, fluoxetine, escitalopram, and sertraline are the preferred drugs [20]. In Australia, the preferred selective serotonin reuptake inhibitors are fluoxetine, sertraline, paroxetine, fluvoxamine, and escitalopram [23]. Studies in Canada and Australia have reported various antidepressants under the serotonin-norepinephrine reuptake inhibitors [20,23].

A qualitative analysis of the trend of antidepressant use in our review exhibited fluctuations over the years. We observed that in 2013, the range was 0.85% to 20.9% [18,20,21,23]. In 2014, there was a slight increase, with a range of 0.90% to 22.3% [18,20,21,23,25]. Subsequently, in 2015, there was a substantial rise in antidepressant use in six studies, ranging from 1% to 44.9% [18-21,23,25]. However, in 2016, the rate decreased to a range of 1.07% to 24.4% [20,21,23,25]. In 2017 and 2018, the rates were reported as 25.5% and 24.2%, respectively [23]. The use of antidepressants varied between 2013 and 2015, decreased in 2016, and then stabilized in 2017-2018.

Anticonvulsants, also known as antiepileptics, are a diverse group of medications that exert their effects through various mechanisms to control seizures. Toxicity associated with antiepileptic use typically manifests as a triad of symptoms, including central nervous system (CNS) depression, ataxia, and nystagmus [40]. In a study conducted among individuals with autism, the prevalence of anticonvulsant use was reported as 14% in each of the years from 2013 to 2016 [25].

Another class of drugs examined in the included studies was anxiolytics, which are prescribed for the treatment of panic disorders, generalized anxiety, and other related conditions [41,42]. A small proportion of the population studied was using anxiolytics for the treatment of autism, and major depressive disorders. Among the included studies, the prevalence of anxiolytics use range was 0.14% to 3% in 2013 [21,23,25], 0.15% to 2.9% in 2014 [21,23,25], 0.14% to 3.1% in 2015 [21,23,25], and 0.15% to 3% in 2016 [21,23,25]. One study mentioned that the prevalence rates of anxiolytic use were 3.3% in 2017 and 2.5% in 2018 [23]. The prescribed anxiolytics in Australia and New Zealand were diazepam, clobazam, oxazepam, alprazolam, and lorazepam [21,23].

Sedatives that have amnestic properties are preferable as they can help to prevent or alleviate anxiety and agitation [32]. In New Zealand and Australia, hypnotics/sedatives were commonly used to treat tension and anxiety. Midazolam, nitrazepam, temazepam, and zopiclone were the most commonly used drugs in these countries. Additionally, lormetazepam, phenobarbitone sodium, and triazolam were also used in New Zealand [21,23]. Additionally, melatonin was used as a hypnotic for disorders not only in New Zealand and Australia but also in the United States [21,23,26].

Limitations

It is important to acknowledge the limitations of this systematic review. The included studies may have variations in methodologies, sample sizes, populations, and indications of psychotropic medications, which could affect the findings. The review focused on trends and prevalence rates without examining specific factors influencing prescribing patterns. Additionally, the review did not assess long-term outcomes or evaluate the appropriateness and effectiveness of psychotropic medication use.

This review has a significant implication on psychotropic medication uses in children and adolescents from 2013 to 2023, which informs healthcare providers, policymakers, and researchers about medication trends and shifts in prescribing practices. It identified research gaps and contributed to clinical practice guidelines.

## Conclusions

Psychotropic medications have been frequently used in children and adolescents with mental health problems between 2013 and 2022. Antipsychotic prescribing has declined in recent years, while stimulants have remained consistent. Antidepressant use has fluctuated, with a significant increase in 2015, followed by a decrease in subsequent years. Anticonvulsants and anxiolytics were also used but at lower rates. Further research is needed among similar populations to understand the reasons behind these trends and to evaluate the long-term effectiveness and safety of psychotropic medications in children and adolescents.
